# Mechanism of lung development in the aetiology of adult congenital pulmonary airway malformations

**DOI:** 10.1136/thoraxjnl-2020-214752

**Published:** 2020-07-30

**Authors:** Bethany Taylor, Alexandra Rice, Andrew G Nicholson, Matthew Hind, Charlotte H Dean

**Affiliations:** 1 National Heart and Lung Institute, Imperial College London, London, UK; 2 Department of Histopathology, Royal Brompton and Harefield NHS Foundation Trust, London, UK; 3 Respiratory Medicine, Department of Respiratory Medicine and National Institute for Health research Respiratory Biomedical Research Unit at the Royal Brompton NHS Foundation Trust and Imperial College, London, UK

**Keywords:** airway epithelium, lung cancer, TTF-1

## Abstract

Congenital pulmonary airway malformations (CPAMs) are rare lung abnormalities that result in cyst formation and are associated with respiratory distress in infants and malignant potential in adults. The pathogenesis of CPAMs remains unknown but data suggest disruption of the normal proximo-distal programme of airway branching and differentiation. Here, we demonstrate that adult human CPAM are lined with epithelium that retains SOX-2 and thyroid transcription factor-1 immunohistochemical markers, characteristic of the developing lung. However, RALDH-1, another key marker, is absent. This suggests a more complex aetiology for CPAM than complete focal arrest of lung development and may provide insight to the associated risk of malignancy.

## Introduction

The three-dimensional network of conducting airways is formed during the pseudoglandular phase of lung development, by branching morphogenesis. This process is controlled by temporal and spatial localisation of key genes and signalling molecules that co-ordinate to drive organ formation. The fundamental pathways used during development also have key roles in tissue haeomeostasis and repair once the adult lung is formed; dysregulation of these same developmental pathways contributes to the pathogenesis of many adult diseases including emphysema,^1 2^ fibrosis^3^ and lung cancer.^4^

Congenital pulmonary airway malformations (CPAMs) comprise a spectrum of lung abnormalities estimated to affect between: 1:11 000 and 1:35 000 live births.^5^ CPAMs are associated with neonatal respiratory distress, recurrent childhood pulmonary infection and, without resection, malignant potential in adults.^6 7^ CPAMs have traditionally been classified into types 0–4 depending on size and location of lesions present. The most common sub-types are 1 (60%–70%), characterised by large (>2 cm) intercommunicating cysts, and 2 (15%–20%), consisting of small (<2 cm) cysts and solid tissue^8^

The high mobility group box transcription factor SOX-2, early embryogenic transcription factor thyroid transcription factor-1 (TTF-1) and the retinoic acid synthesising enzyme RALDH-1 are necessary for normal patterning and development of airway branching in the mouse lung.^9^ In addition, SOX-2 overexpression in mouse has been previously found to result in cystic lesions resembling those observed in human CPAM.^10^ It is therefore hypothesised that alterations in signalling during branching morphogenesis underlie CPAM pathogenesis. In these experimental pathology studies, we examine localisation of the proteins TTF-1, SOX-2 and RALDH-1 in lung tissue from 14 cases of type 1 and 2 cases of type 2 adult CPAM compared to normal adult lung parenchyma and embryonic (fetal) human lung.

## Methods

Fourteen type 1 and 2two type 2 CPAM resection specimens were obtained from the diagnostic archive of the Royal Brompton Hospital (ethics reference number 10/H0504/9). Normal adult lung tissue was obtained from the International Institute for the Advancement of Medicine (USA). Human fetal lung sections (14 weeks post conception, WPC) were provided by the Joint MRC/Wellcome Trust (099175/Z/12/Z) Human Developmental Biology Resource (www.hdbr.org). Sections of adult normal resected, CPAM and fetal human lung tissue underwent immunohistochemical (IHC) staining for SOX-2, TTF-1 and RALDH-1. Sections were incubated with primary antibody at optimised concentrations (Dako TTF clone 8G7G3/1 (M3575) at 1:500, Immune Systems. SOX-2 (GT15098) at 1:500 and Sigma ALDH1A1 (HPA002123) at 1:250) overnight at 4°C. Sections were then incubated with biotinylated antibody (1:50) from the VectaStain ABC kit followed by ABC reagent (1:50) and DAB substrate application. Sections were mounted in DPX and digital images were captured at 20× magnification using Leica Application Suite software (Leica microsystems, Milton Keynes, UK) for qualitative comparison of lung tissue sections.

## Results

### SOX-2

**Figure 1:**
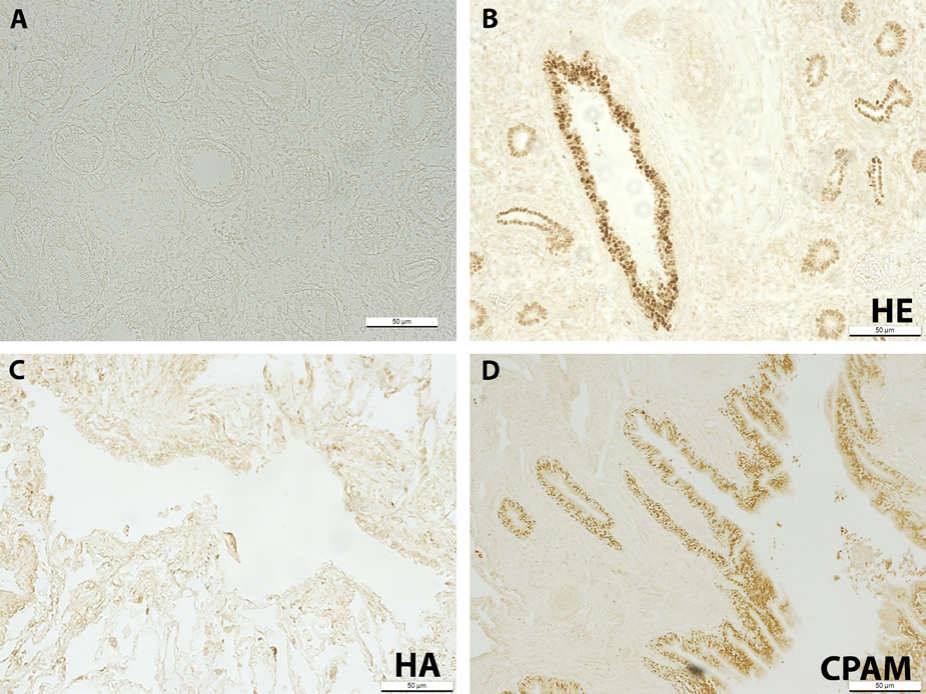
Fourteen WPC normal human embryonic lung with primary antibody omitted (negative control) (A); SOX-2 immunostaining in 14 WPC normal HE lung (B); normal HA lung (C) and CPAM HA lung (D). Scale bar, 50 µm. CPAM, congenital pulmonary airway malformation; HA, human adult; HE, human embryonic; WPC, weeks post conception.

Control embryonic lung section with primary antibody omitted shows no positive staining (figure 1A). In comparison, embryonic lung tissue shows positive SOX-2 immunostaining localised to airway epithelial cells with strong staining present in the large, proximal airways (figure 1B). SOX-2 was absent in normal adult epithelium (figure 1C) whereas positive SOX-2 staining was observed in CPAM lung tissue sections, localised to cyst-lining epithelial cells (figure 1D).

### TTF-1

**Figure 2:**
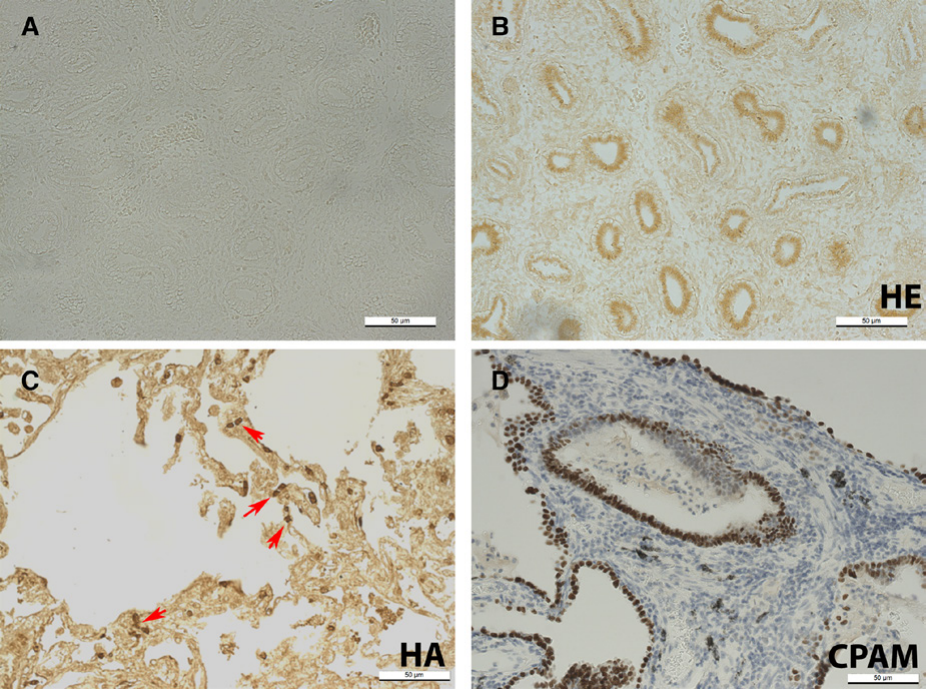
Fourteen WPC normal human embryonic lung with primary antibody omitted (negative control) (A); TTF-1 immunostaining in 14 WPC normal HE lung (B); normal HA lung (C) and CPAM HA lung (D); counterstained with haemotoxylin (D). Red arrows in (D) indicate positive cells. Scale bar, 50 µm. CPAM, congenital pulmonary airway malformation; HA, human adult; HE, human embryonic; WPC, weeks post conception.

Control embryonic lung section with primary antibody omitted shows no positive staining (figure 2A). TTF-1 was localised to airway epithelial cells in embryonic lung tissue (figure 2B), but in adult sections was present in a sub-set of alveolar cells (figure 2C). In CPAM lung tissue sections, all cyst lining epithelial cells stained positively for TTF-1 (figure 2D).

### RALDH-1

**Figure 3:**
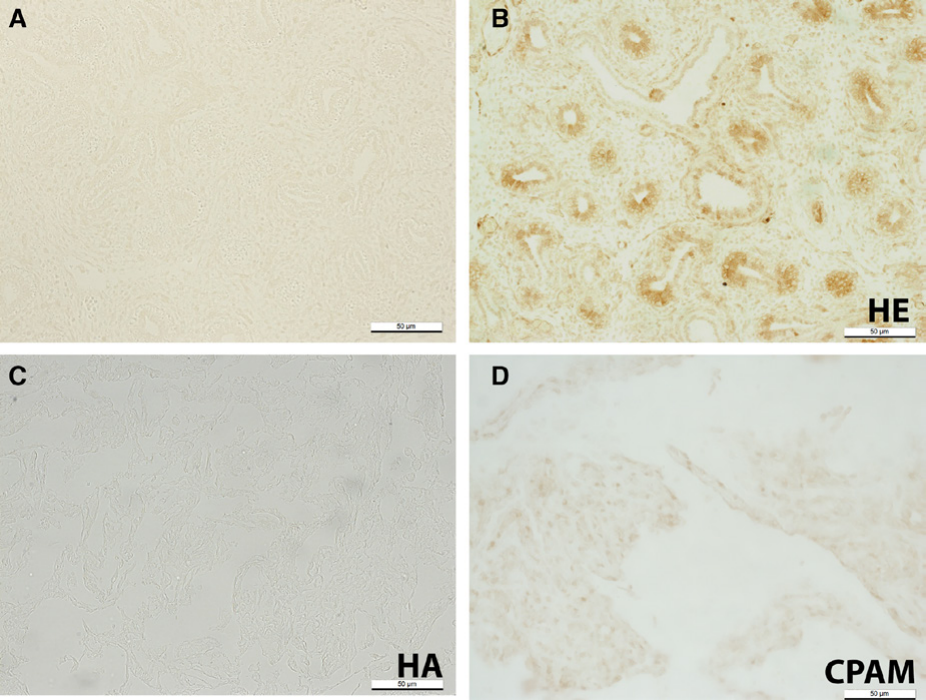
Fourteen weeks post conception (WPC) normal human embryonic lung with primary antibody omitted (negative control) (A); RALDH-1 expression in 14 WPC normal HE lung (B); normal HA lung (C) and CPAM human adult (HA) lung (D). Scale bar, 50 µm. CPAM, congenital pulmonary airway malformation; HA, human adult; HE, human embryonic; WPC, weeks post conception.

Control embryonic lung section with primary antibody omitted shows no positive staining (figure 3A). Positive staining was localised to the airway epithelium in embryonic lung tissue (figure 3B). RALDH1 staining was absent in normal adult lung tissue (3K). Similarly, RALDH1 staining was absent, or only very weakly positive in CPAM lung tissue sections (figure 3C).

## Discussion

This study shows that the embryonic airway epithelial cell markers SOX-2 and TTF-1 are retained in the cyst-lining epithelial cells of CPAM human lung tissue. However, localisation of another key development protein, RALDH1, reported here for the first time, is similar to that of adult lungs. This suggests that the phenotype of CPAM epithelial cells may be more complex than complete focal arrest in development.

SOX-2, TTF-1 and RALDH1 expression in normal adult and embryonic lung tissue sections, as represented by positive IHC staining, was consistent with previous studies.^10 11^ SOX-2 is critical to cell pluripotency and self-renewal, with principal embryonic expression in the proximal airway epithelium during bronchiole development. In pseudoglandular human lungs, SOX-2 levels are high in proximal stalk epithelium (differentiating bronchioles), with low levels of SOX-2 present in the distal tip epithelium. Tip expression of SOX-2 disappears completely by 20 WPC and, as a result, involvement in peripheral alveolar development is minimal, and SOX-2 protein is absent in the adult lung.^12^ However, expression was present in the cyst-lining epithelial cells of adult CPAM sections, suggesting cystic regions retain a proximal embryonic phenotype. This is supportive of previous studies by Ochieng *et al* demonstrating the likely role of SOX-2 overexpression in CPAM cyst development.^10^

TTF-1 expression in adult human lung tissue was localised to a sub-set of alveolar epithelial cells, presumably type II pneumocytes and absent from airway epithelium. TTF-1 is localised to all airway epithelial cells in human embryonic lungs but unlike SOX-2, there is no difference between proximal and distal airways. TTF-1 staining in cyst-lining epithelium of CPAM lung tissue more closely resembled the generalised epithelial expression of embryonic lung tissue.

RALDH1 expression was prominent in airway epithelial cells of pseudoglandular lung, whereas no staining was present in the adult lung. In CPAM lung tissue sections, some extremely weak RALDH1 staining was observed when compared with normal adult tissue but the pattern of staining did not resemble that seen in embryonic sections, suggesting that the phenotype of cyst-lining epithelium in CPAM differs from that of embryonic lung epithelium.

These findings may also be relevant to the unusual association of lung cancer developing in some CPAMs, mainly type 1 and rarely type 2.^5 13^ Both *SOX-2* and *TTF-1(NK2X-1*) genes are associated with tumour plasticity^14^ and a decrease in expression of genes that regulate retinoid metabolism and signalling, including RALDH1, has also been reported in non-small cell lung cancers.^15^ Although most cancers associated with type 1 CPAMs are mucinous adenocarcinomas^5 13^ and therefore negative for TTF-1, persistence of SOX-2 expression and loss of RALDH1 in the epithelial cells may have some bearing on tumour development and warrant further study.

We recognise that this paper has limitations, in that our cohort is limited to types 1 and 2 CPAMs. However, current thinking is that there are greater similarities between large cyst type (Stocker type 1) and the small cyst type (Stocker type 2) CPAMs, with the remaining three morphologic types believed to be different pathogenetic processes, namely acinar dysplasia (type 0), pulmonary hyperplasia (type 3) and low-grade cystic pleuropulmonary blastoma (type 4).^16^

In conclusion, our data show novel variations in embryonic airway epithelial cell markers that will hopefully lead to improved understanding of CPAM development.
